# Factors impacting the delivery of contextualized care in serious illness: a focus group study with healthcare professionals

**DOI:** 10.1186/s12916-026-04662-w

**Published:** 2026-01-29

**Authors:** Linda Modderkolk, Yvonne Schoon, Hugo Touw, Yvonne Engels, Anne B. Wichmann

**Affiliations:** 1 Department of Anesthesiology, Pain and Palliative Medicine, Radboudumc, Nijmegen, the Netherlands; 2 Department of Geriatrics, Radboudumc, Nijmegen, the Netherlands; 3 Department of Intensive Care Medicine , Radboudumc, Nijmegen, the Netherlands

**Keywords:** Serious illness, Palliative care, Patient-centred care, Contextualized care, Patient context, Implementation science, Theoretical Domains Framework

## Abstract

**Background:**

As the number and complexity of patients living with serious illness continue to rise, delivering care that is both effective and responsive to individual life contexts has become increasingly important. Despite its potential benefits, the implementation of contextualized care in the management of serious illness remains limited and poorly understood. To address this gap, this study aimed to identify barriers and facilitators influencing the delivery of contextualized care for patients with serious illness, as perceived by healthcare professionals (HCPs), and to generate recommendations for improving its implementation.

**Methods:**

Three focus groups were conducted with 20 HCPs from multiple disciplines and hospital settings in the Netherlands, all involved in the care of patients with serious illness. Discussions were guided and analysed using a directed content analysis informed by the COM-B model (Capability, Opportunity, Motivation–Behaviour) combined with the Theoretical Domains Framework. Factors were mapped to intervention functions from the Behaviour Change Wheel (BCW) to provide recommendations.

**Results:**

Nine factors influencing contextualized care were identified across COM-B components. Capability-related factors included skills and knowledge to engage with the relevant patient context and the ability to distinguish between general and clinically relevant context. Opportunity-related factors included environmental conditions, fragmented information systems, systemic incentives misaligned with contextual care, a lack of shared team norms, collaboration challenges, and the perceived emotional complexity of contextual conversations in the palliative phase. Motivation-related factors included strong intrinsic commitment to person-centred care and awareness of the consequences of overlooking context for patients, HCPs, and the overall system. Most barriers were concentrated in the *Opportunity* component, with environmental and team-level constraints often outweighing individual motivation and basic skills.

**Conclusions:**

Delivering contextualized care for patients with serious illness is not primarily limited by individual willingness or basic capability but by environmental and systemic feasibility. Sustainable implementation requires multilevel strategies targeting team culture, interprofessional collaboration, and a supportive infrastructure. Moving from individual intent to shared norms may improve both patient outcomes and resource efficiency. Key steps include continuous education, embedding contextual care in team culture, adapting workflows and documentation, and integrating contextualization into quality measures and incentives.

**Supplementary Information:**

The online version contains supplementary material available at 10.1186/s12916-026-04662-w.

## Background

A growing number of patients are living with serious illness: conditions characterized by a high risk of mortality and a significant impact on their quality of life and daily functioning [1]. Palliative patients form a subgroup within this broader serious-illness population, although “palliative” is frequently interpreted narrowly by healthcare professionals (HCPs) as referring only to end-of-life care. In reality, complex care needs can arise at any stage of serious illness, as these conditions often affect multiple dimensions of a patient’s life. With the increasing prevalence of multiple chronic conditions (MCC), such needs may emerge earlier in the disease trajectory, complicating care planning well before the end of life [2]. Therefore, in recent decades, the care for these patients has become increasingly specialized and complex, driven by technological advancements and the rise in multimorbidity. As clinical complexity increases, delivering care that is both effective and aligned with patients’ lived realities becomes more challenging [2–4]. This calls for contextualized care: an approach in which HCPs actively identify and integrate relevant life context — such as social, psychological, or economic factors — into medical decision-making and care planning [5].

Contextualized care is closely related to approaches like person-centred or holistic care, which aim to personalize treatment choices based on a patient’s preferences, values, and needs. However, it differs in its explicit focus on integrating contextual factors — such as a patient’s living situation or caregiving responsibilities — that determine whether a care plan is realistically feasible in daily life and clinically appropriate. Particularly in serious illness, where healthcare costs are high and treatment burden is significant, contextualized care may help avoid overtreatment, reduce unnecessary interventions, and enhance outcomes that matter to patients [6–14].

To support contextualized care in practice, HCPs often rely on shared decision-making (SDM) and advance care planning (ACP). SDM facilitates the best decision-making by incorporating medical information and patient preferences through open dialogue [15, 16]. ACP, in turn, is defined as the process of enabling individuals to define goals and preferences for future medical treatment and care, to discuss these with family and HCPs, and to record and review them when appropriate [17, 18]. While both approaches aim to centre care around the individual, they often insufficiently address the broader context of patients’ lives [19]. For example, a cancer patient eligible for palliative chemotherapy who also cares for a spouse with dementia may require a different treatment plan, as their caregiving responsibilities should be taken into account when determining the most appropriate option. Integrating contextual factors can help align medical care with patients’ day-to-day realities, improve care quality, and prevent misuse of resources [20].

Despite growing recognition of its importance, the systematic incorporation of patient context into clinical decision-making remains limited in both training and practice, even in serious illness. Educational curricula increasingly emphasize person-centred care yet often focus primarily on relational and communication skills, with less attention to contextual data gathering and application [21–24]. Moreover, structural challenges—such as persistent staff shortages, rising healthcare costs, increasing technological options, and a reluctance to initiate timely end-of-life discussions—further complicate the delivery of contextualized care [6, 7, 25–28]. To effectively design and implement interventions that support this type of care, a better understanding of the practical barriers and facilitators is needed [29].

Although previous work has described the concept of contextualized care and documented the consequences of contextual errors, little is known about why contextualization remains difficult to implement in real-world clinical practice. To our knowledge, no prior qualitative study has systematically examined the behavioural, team-based, and system-level determinants of contextualized care using established implementation science frameworks. Moreover, contextualized care has received little explicit attention within the serious-illness literature, despite its clear relevance for guiding appropriate clinical decision-making in these settings. This focus group study therefore aimed to identify the key factors that influence the delivery of contextualized care for patients with serious illness, as experienced by healthcare professionals in the Netherlands, and to provide recommendations for improvement.

## Methods

### Study design

A focus group study was conducted to (1) identify key factors that influence the delivery of contextualized care for patients with a serious illness by HCPs and (2) provide recommendations for interventions to support this process. The study was guided by three interconnected frameworks: the COM-B (Capability, Opportunity, Motivation–Behaviour) model, the Theoretical Domains Framework (TDF), and the Behaviour Change Wheel (BCW) [30–32].

### Theoretical framework

**Fig. 1:**
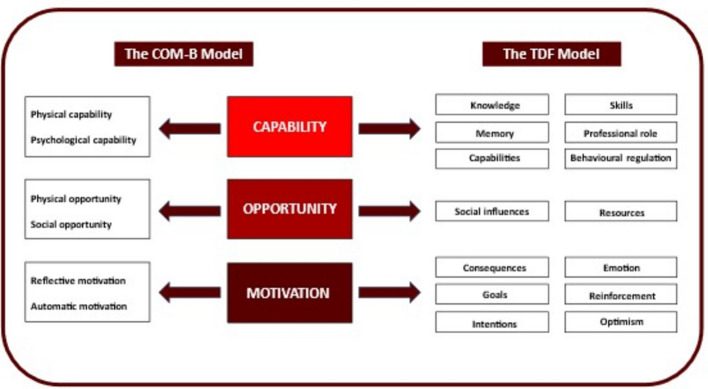
The combined COM-B/TDF model

In this study, we focused on the behaviour of HCPs’ signaling, exploring, and integrating the life context of patients with a serious illness into care provision and planning. Following the method proposed by Atkins et al. [33], we linked the COM-B components with the 14 TDF domains, forming an integrated COM-B/TDF framework (see Fig. 1). These frameworks were selected because they offer a structured and theory-driven approach to identifying determinants of behaviour and translating them into intervention strategies, which aligns closely with the aim of this study.

#### COM-B model

To identify factors that impact HCPs behaviour, we used the COM-B model and the TDF.

The COM-B model suggests that behaviour (B) is influenced by three key factors: Capability (C), which includes skills and knowledge, Opportunity (O), meaning the surrounding environment that enables or hinders behaviour, and Motivation (M), a person’s drive or willingness to act [31, 33].

#### Theoretical domains framework

The TDF further breaks down these components into 14 specific domains that influence behaviour [32, 34]. These domains help identify more specific factors that shape HCPs’ actions, especially when adopting new approaches.

#### The behaviour change wheel

**Fig. 2:**
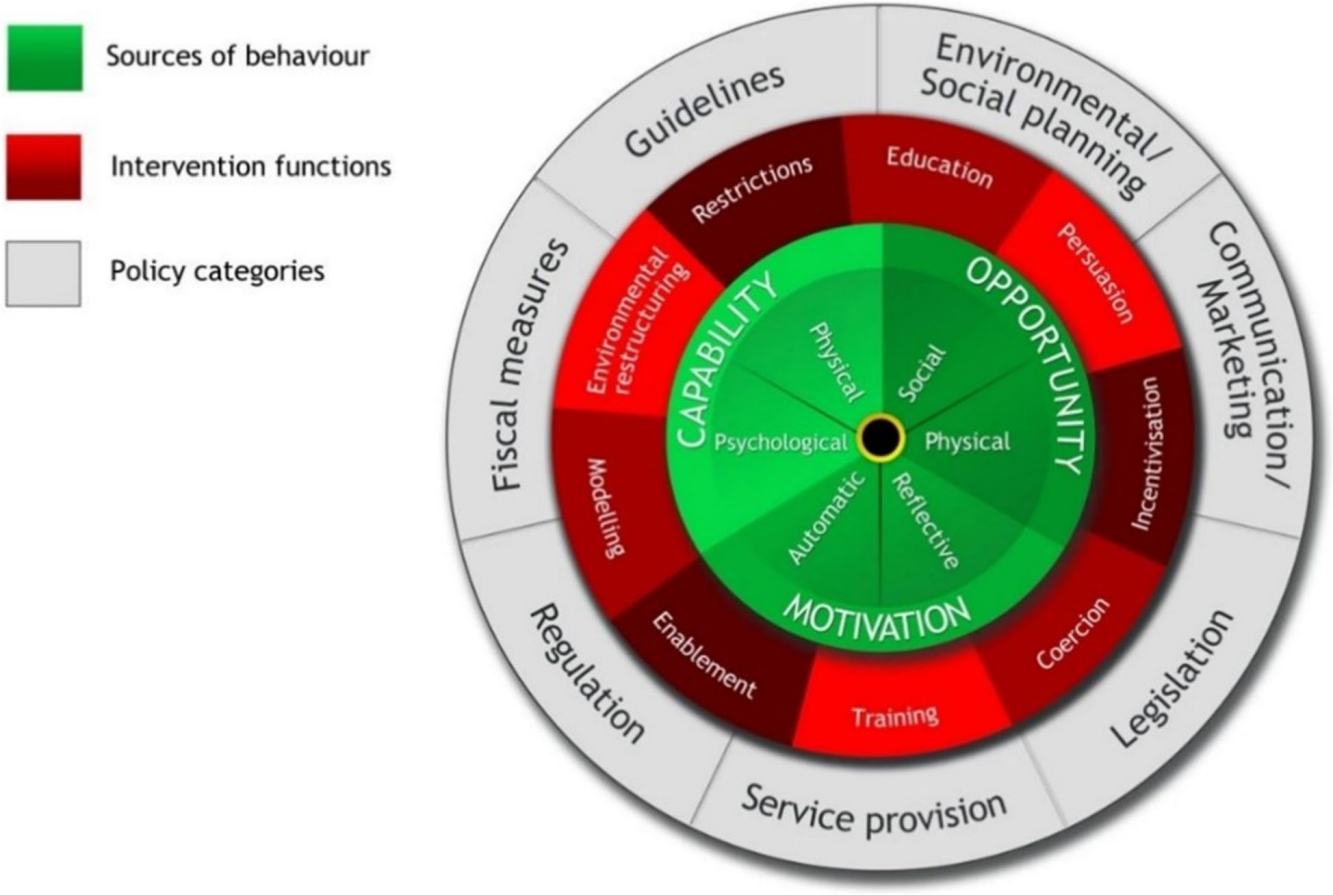
The Behaviour Change Wheel (Michie et al., 2011) [31]

To translate these behavioural factors into actionable interventions, we applied the BCW [31]. This framework links elements of the COM-B model to nine intervention functions: education, persuasion, incentivization, coercion, training, restriction, environmental restructuring, modeling, and enablement. It also considers relevant policy categories that can facilitate or constrain these interventions (see Fig. 2.) We used the BCW to formulate possible interventions based on the factors found to be related to the COM-B components.

### Participants and sample size

The focus groups were open to HCPs who work in a Dutch hospital team and provide care for patients with a serious illness. We aimed to include physicians, nurses, nursing specialists, and physician assistants. Recruitment was conducted using a convenience sampling method, inviting HCPs through the researchers’ network using a snowball sampling approach. We planned to perform three focus groups, each with 6–10 participants. Two of them were scheduled to take place in an academic hospital and one in a peripheral hospital. We aimed to include a diverse group of participants based on age, gender identity, discipline, specialism, years of work experience, hospital type, and affinity with the topic. We aimed for thematic saturation, which refers to the point at which no new themes or insights emerge from the data [35, 36]. Our sample size aligns with established qualitative research guidelines, which suggest that 3–6 focus groups are typically sufficient to achieve saturation in studies with relatively homogenous participants [35, 37–39]. To be eligible, participants had to work in a hospital team, be actively involved in caring for patients with a serious illness, be fluent in Dutch, be 18 years or older, and provide informed consent.

### Data collection

Live focus group meetings in two hospitals were held in March 2024, each conducted by two researchers (L. M. and A. B. W./Y. E.). One researcher acted as the lead moderator, guiding the discussion, while the second researcher served as an observer and note taker, documenting key points on a flip chart and posing occasional follow-up questions to ensure depth and clarity. The meetings lasted 90 min and were audio-recorded. Verbatim transcriptions were made, ensuring the anonymity of HCPs and patients. A brief presentation preceding the discussion introduced participants to Weiner’s theory and the contextual domains to clarify the concept of contextualization of care and relevant patient context [5]. The focus group centred around the target behaviour mentioned in the theoretical framework, and the guide for the focus group can be found in Supplement 1.

To mitigate potential power dynamics within the mixed-discipline groups—such as nurses and physicians—we set ground rules emphasizing confidentiality, the value of diverse perspectives, and explicit encouragement of differing viewpoints. Participants were first invited to write down initial reflections individually, and throughout the session, the moderator checked whether all perspectives, including minority or divergent ones, had been voiced and that all participants had given input. To further support safety, participants were also given the opportunity to indicate in advance if they preferred not to be placed in a focus group with direct colleagues. We used follow-up probes and questions to gain deeper insights into the relevant domains related to the target behaviour of contextualizing care for patients with a serious illness. Following the focus groups, transcripts were returned to the participants for member checking, allowing them to correct or add insights via email.

### Data analysis

A directed content analysis was employed to define and distinguish relevant domains within the COM-B/TDF related to the specified target behaviour [40]. The researchers formulated operational definitions for each TDF domain to examine their relevance to the contextualization of care in clinical practice (see Supplement 2). Subsequently, the first focus group was read and coded independently by two researchers (L. M., A. B. W.) using Atlas.ti 23.1.1 software, and the coding was discussed until consensus on the codebook was achieved. One of the researchers (L. M.) then continued to analyse the other two focus groups. Frequency-based descriptions were used to offer an initial understanding of the data by highlighting the most frequently discussed TDF domains. Following this step, overarching themes within the TDF domains were elicited and, as a final step, analysed within the COM-B model. Lastly, findings were synthesized into nine overarching factors, and their interconnected dynamics were highlighted. A peer session was organized to discuss the identified factors and interconnections (L. M., Y. S., H. T., Y. E., A. W.). Across the three focus groups, thematic saturation was reached: the third focus group contributed only minor refinements to existing codes and did not introduce any additional themes or TDF domains, indicating that further data collection was unlikely to yield new insights. Subsequently, the research team formulated recommendations by linking the identified factors to relevant intervention types as described by the BCW. Findings are reported following the consolidated criteria for reporting qualitative research (COREQ) [41] (see Supplement 3).

### Research team and reflexivity

L. M. is a female PhD student and counsellor with extensive experience in qualitative research. Y. E. is a female professor of meaningful healthcare. H. T. is a male intensivist at an academic hospital and a researcher. Y. S. is a female professor of appropriate care and a geriatrician in an academic hospital. ABW is a female assistant professor in palliative and contextual care. Aware of how our backgrounds could influence interpretation, we reflected regularly during biweekly team meetings, questioned assumptions, and grounded our analysis in participants’ own words. Because recruitment occurred partly through professional networks, some participants were acquainted with one or more researchers. To minimize potential influence on participants’ contributions, this was openly acknowledged at the start of each focus group, and moderators emphasized confidentiality, the value of diverse perspectives, and the importance of freely expressing differing viewpoints.

## Results

### Participants

Three focus groups were conducted, including a total of 20 participants (16 women, 4 men). Participants consisted of physicians, nursing specialists, nurses, and a physician assistant, representing 14 different specialisms. Participant characteristics can be found in Table 1.

**Table 1:** Characteristics of focus group participants

Characteristic	Participants (number)
**Gender identity (*n*)**	
Women	16
Men	4
**Age (*n*)**	
20–29 years	1
30–39 years	3
40–49 years	6
50–59 years	7
60–69 years	3
**Specialism (*n*)**	
Geriatrics	3
Anaesthesiology	2
Cardiology	2
Intensive care	2
Pulmonology	2
General practice	1
Gynaecology	1
Haematology	1
Internal medicine	1
Nephrology	1
Oncology	1
Oral and maxillofacial surgery	1
Palliative care	1
Surgery	1
**Discipline (*n*)**	
Physician	9
Nursing specialist	8
Nurse	2
Physician assistant	1
**Years of work experience (*n*)**	
0–9 years	2
10–19 years	5
20–29 years	3
30–39 years	6
40–49 years	4
**Hospital type (*n*)**	
Academic hospital	15
Peripheral hospital	5
**Self-rated affinity with contextualizing care (1–10 scale) (*n*)**^**a**^	
1–2	0
3–4	0
5–6	1
7–8	6
9–10	11

^a^Not all participants answered this question

### Descriptives

During the focus groups, a total of 265 references were made to factors influencing contextualization of care by HCPs for patients with a serious illness. These references were found across all COM-B components, which span all TDF domains, and they were approximately evenly distributed across the COM-B components. However, while the overall number of references was similar across components, the variation within these references was considerable: Capability- and Motivation-related references tended to reiterate a small set of recurring factors, whereas the Opportunity component encompassed a much broader range of distinct factors. All references were combined to describe nine key factors influencing contextualization of care by HCPs for patients with a serious illness. Illustrative quotes for all factors can be found in Table 2.

**Table 2:** Quotes illustrating the different factors

Factor	Quotes
1. **Skills and knowledge to engage with patient context**	“I think it’s always important to learn to recognize certain cues, because with experience and insight, that puts you several steps ahead. It helps you gain trust, and it gives you a better understanding of the illness and everything surrounding it” “Something else just came to mind: knowledge of other cultures. We're moving toward a multicultural society, and often you catch yourself thinking, ‘Oh, I know what’s best for you.’ But then you realize — for example — that a patient doesn’t want morphine, and you wonder why. […] And also people with lower levels of education or low literacy. Some of them really don’t understand what you’re saying. They respond to the doctor with ‘yes, sure,’ but they’ve actually understood nothing. If you’ve never been to a hospital before and are suddenly confronted with treatment — and everything is moving so fast — you might not understand it at all. And if you also struggle to read or write, then you can’t just go back and reread the information. That’s a cultural issue, too”
2. **Supportive environment for contextual conversations**	“There are all sorts of things on my mind that go beyond just time — and maybe it’s related — but it’s about being able to give attention. That I can be fully present, not distracted by a thousand other things around me, but truly in the here and now, for this man or woman sitting in front of me” “And when you schedule a longer conversation, you hand over your pager, put your phone away — so you’re not getting called in the middle of what is meant to be a meaningful conversation”
3. **Fragmented information systems hinder contextual care**	“Well, we’re in a process where the electronic health record really isn’t helping. We don’t know where to document this kind of information, and we also can’t share it directly with primary care. So we end up needing all sorts of workarounds — logging into different systems — and that’s really a barrier”
4. **Systemic drivers that misalign with contextual care**	“But also the financial structure of the hospital — like you [*other HCP*] said: *not* treating someone actually costs money. And it’s the same with future care planning, right? Technically, we can already code those conversations in a way that they’ll be reimbursed in the future. Right now, there’s still no payment for them. But then I heard that even if you do book those conversations and write your report, the health insurer apparently requires an MDT [*multidisciplinary team meeting*] to have taken place as well. And then I think — how absurd is that? Why? An MDT should only happen when there are uncertainties, not when everything is already clear. That just doesn’t make sense”
5. **Lack of shared team norms around contextual care**	“For example, when you’re working towards the end of life with a patient, and then a medical oncological treatment is introduced [*by another professional],* you move further away from that phase. Important and timely conversations about the end of life are then postponed, even though they’re still very relevant” “It also really depends on the individual whether they find this important. There are plenty of people who are highly motivated and skilled on a technical or surgical level, but who think, ‘Ugh, do we really have to go gather all that information from the patient?’ That’s why it’s so important to divide tasks well within the team and complement each other”
6. **Collaboration and communication across healthcare professionals**	“A sense of trust in the whole team — that when things are documented, they’ve also been properly discussed, even if they’re not written in the family notes. That there’s a good handover, and that information carries over properly from one provider to the next” “Lack of familiarity with collaboration: in the hospital, we operate on an island — both toward the outside world and even within the hospital itself. Even within our own specialty, we often don’t take a moment during ward rounds to make a quick call like, ‘Hey, you’re the regular contact for this patient — could you think along with us?’ Even that seems beyond us. We simply don’t know how to find each other”
7. **Trust and emotional complexity in patient interactions**	“And I think there’s another layer to this: people can sometimes react quite strongly or impulsively — even somewhat aggressively — saying things like, ‘Well, that’s my decision,’ or, ‘I just want this.’ They may come in with very specific demands. In my experience, many of these demands come from things they’ve looked up themselves — things they’ve Googled — which often make little sense or lack any real grounding. It really hampers meaningful conversation” “There’s also pressure from family — I had written that down under factors that influence a person’s choices. Family systems and relatives can have a huge impact. That makes conversations about not treating someone really difficult for a physician, especially when you only have 15 min. It’s almost impossible”
**8. Intrinsic motivation to provide person-centred care**	“People who work in healthcare — and I’m not sure if I should generalize — but we genuinely enjoy working with people. So I believe that intrinsic motivation is something we all share. We truly want to understand the person sitting in front of us” “I also have a bit of self-interest. Exploring the context of the patient is what makes my profession enjoyable. Every day is different, and every day brings a new surprise. That makes me happy. If I weren't allowed to do that, I would feel like I'm working in a factory. It’s what makes our profession both enjoyable and special. Being able to do this is what enables me to sustain myself in this profession.”
9. **Consequences of overlooking or integrating patient context**	“So often we’ve had cases where someone spent a month in the ICU, went through a long and intensive process, and in the end the patient says, ‘I didn’t even want any of this.’ Of course, you can’t always know that in advance — but it’s really a shame. We’ve put someone through all that, thinking: ‘Well, they survived the ICU, we did a good job.’ But the patient saw it completely differently. It’s about the value *for the patient* — and that’s what we’re missing” “I can also imagine that some people [HCPs] just see it as a hassle — like, ‘This leg just needs to heal, so get out of my way, I’m busy.’ For me, a successful outcome is one with as little trouble afterward as possible. Because if you don’t assess things properly up front, you’ll have problems later. That’s a big motivation for me too. I think, for example: if I prescribe an inhaler instead of a pill the patient can’t swallow, they’re not going to call me ten times saying they’re short of breath”

### Nine factors influencing contextualization of care

#### Capability

##### Factor 1: Skills and knowledge to engage with the relevant patient context

Most HCPs considered themselves to have a basic insight into their patients’ relevant contextual factors and the communication skills to explore them. Improvement was considered possible, especially around sociocultural aspects. Nurses and nurse specialists emphasized that exploring and integrating relevant patient context are a core part of their training. Across all disciplines, though, many HCPs identified a gap between educational ideals and clinical practice. They highlighted the need for additional training in identifying and addressing contextual factors, treatment-specific considerations, and diversity sensitivity. Some also mentioned emotional discomfort or uncertainty when engaging in conversations involving complex or unsolvable patient situations, such as poor living conditions or financial distress.

#### Opportunity

##### Factor 2: A supportive environment for contextual conversations

HCPs emphasized that attention to the relevant patient context requires the right environmental conditions. Adequate time, quiet settings, and minimal distractions during consultations were mentioned as essential to meaningful patient interactions. Specific practices such as sitting down, turning off phones, and reviewing the patient’s medical record beforehand were seen as supportive strategies, albeit not yet mainstream. At a broader level, system-level constraints—such as limited time, high workload, and staffing shortages—were perceived as significant barriers to contextual care, even when HCPs valued it. HCPs noted that stress in their working environments frequently carried over into their mental states and behaviour, thereby compromising their ability to adequately attend to the patient’s context.

##### Factor 3: Fragmented information systems hinder contextual care

The structure and usability of electronic patient records were frequently mentioned as major barriers to integrating patient context. HCPs expressed frustration with fragmented information, poor interoperability between systems, and the separation between nursing and medical documentation. They also noted inconsistencies in terminology and outdated or irrelevant information. These issues made it difficult to access a coherent picture of the patient, especially in acute situations, and increased the cognitive load on HCPs who had to manually gather and cross-reference contextual information.

##### Factor 4: Systemic drivers that misalign with contextual care

Several participants pointed to structural issues in the healthcare system that work against contextualizing care. From the participants’ overall perspective, financial incentives often favoured continued treatment over careful reassessment, reinforcing a focus on curative pathways or ongoing palliative treatment rather than on appropriate contextualized care. Societal expectations—both from patients and family members—sometimes pressured participants to continue treatments, even when misaligned with the patient’s relevant context. Some also noted that younger or less experienced colleagues were particularly susceptible to these pressures, which could contribute to overtreatment and an unnecessary burden on the system.

##### Factor 5: Lack of shared team norms around contextual care

Despite widespread awareness among participants of the importance of integrating relevant patient context into medical care, especially in the palliative phase, HCPs noted that attention to contextual factors is not yet a shared norm within and across teams. Differences in team culture, professional background, and clinical focus led to inconsistent prioritization of contextualization of care. In some settings, particularly acute environments, biomedical outcomes were prioritized, often at the expense of attention to patients’ broader contextual needs. HCPs experienced this misalignment between and within teams as leading to fragmented care, and they emphasized the need for a collective mindset shift to normalize contextual care in daily practice.

##### Factor 6: Collaboration and communication among healthcare professionals

Effective integration of patient context was seen as highly dependent on collaboration and communication within and across care teams. Whereas the previous factor described how team norms determine whether contextualized care is prioritized in the first place, this factor reflects the communication processes and structures necessary to effectively exchange contextual information across HCPs. Participants valued high-quality reporting, warm handovers, and shared understanding of the patient’s situation. However, they also described barriers such as lack of coordination, unclear responsibilities, and concerns about confidentiality. Several participants mentioned that not knowing each other personally made it harder to reach out, coordinate, or feel confident that contextual information would be acted upon. Hierarchical dynamics sometimes prevented open communication, particularly when nurses felt that physicians did not take their contextual observations seriously. A lack of structured collaboration across care transitions, especially with general practitioners, further hindered the continuity of contextual care. At times, assumptions about other HCPs’ perceived unwillingness to collaborate in contextualizing care for a specific patient, or limited accessibility, hindered participants from reaching out.

##### Factor 7: Trust and emotional complexity in patient interactions

Unlike the previous factor, which concerned professional communication among HCPs, this factor focuses on the relational and emotional dynamics within patient–provider communication. A strong, trust-based relationship with patients was seen as essential for engaging in contextual communication. When patients felt safe and involved—such as through sharing personal concerns or participating in preparatory tools like questionnaires—participants found it easier to tailor care to the individual patient’s context. However, several participants emphasized that these conversations carried an emotional burden. They described this burden as a sense of vulnerability in decision-making, especially when faced with uncertain outcomes or the risk of disappointing patients and families. Some also hesitated to explore patient context in depth, fearing they would uncover issues for which they lacked the time, tools, or authority to address. Others experienced the burden more as moral discomfort when navigating topics such as cultural values, personal suffering, or the limits of medical care. In addition, participants noted that they did not always have the mental space required to engage in such conversations—due to high workloads or personal circumstances—which further complicated their ability to fully connect with patients on a contextual level. These relational dynamics highlight the perceived emotional and ethical demands involved in contextualizing care.

#### Motivation

##### Factor 8: Intrinsic motivation to provide person-centred care

Participants expressed a strong intrinsic drive to deliver high-quality, person-centred care, grounded in a deep sense of professional responsibility. Patient context was considered an essential part of person-centred care. Their approach to patients was described using values such as empathy, engagement, openness, and dignity. Some participants drew from personal experiences, using these reflections to guide their interactions. Many believed that contextualizing care not only improves outcomes but also strengthens the patient-provider relationship. When conversations were successful and led to appropriate, contextualized care decisions, HCPs reported a sense of joy and fulfilment, which in turn enhanced their job satisfaction.

##### Factor 9: Consequences of overlooking or integrating relevant patient context

While this study focused on HCPs’ perspectives, many participants reflected on the direct consequences for patients when contextual factors are overlooked or not properly addressed. HCPs emphasized that when the patient context is actively considered, care becomes more appropriate, treatment decisions are better aligned with patients’ abilities and preferences, and potential barriers are identified earlier, leading to smoother care trajectories and fewer complications. In contrast, when relevant context is missed or not shared among professionals, participants reported that patients often experience care as fragmented or disjointed. They may encounter repeated questioning, inconsistent decisions, or treatment plans that do not fit their daily realities. Participants highlighted that this not only increases the emotional and practical burden for patients but also leads to reduced trust, poor treatment adherence, and inefficient use of time and resources. Several participants described this as a breakdown in continuity, where care becomes a series of isolated moments instead of a coherent, person-centred process.

### Interconnected factors across individual, team, and system levels

**Fig. 3:**
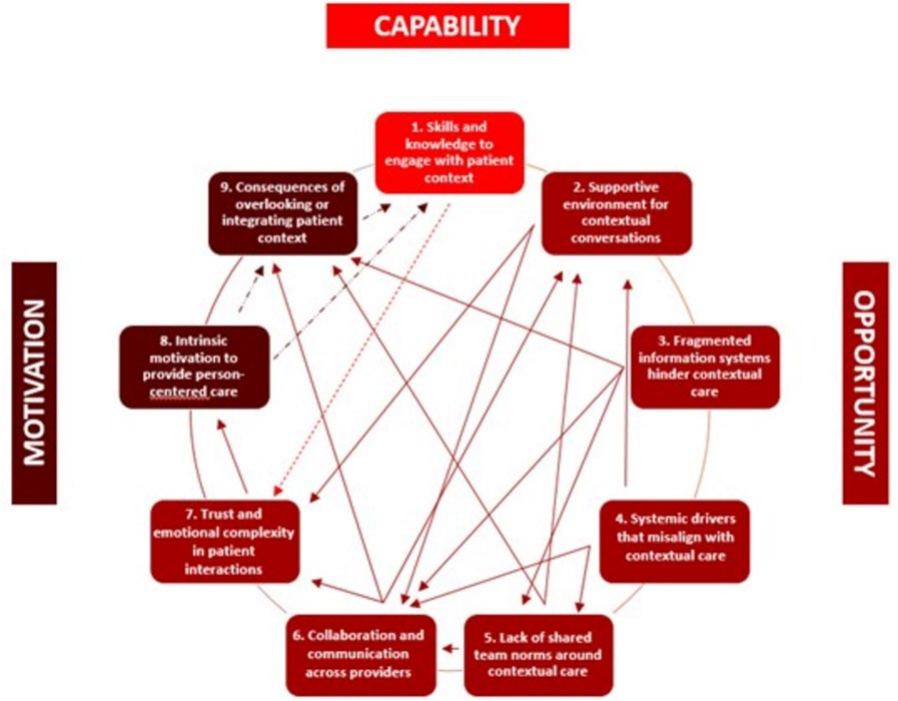
Interconnected factors across COM-B components

This study shows that barriers and facilitators to contextualizing care for patients with serious illness are not isolated at a single level. Rather, they emerge from dynamic interactions between individual HCPs, their teams, and the broader healthcare system, as visualized in Fig. 3. For example, while individual participants were intrinsically motivated to consider patient context, their ability to act on this motivation is often shaped by the norms within their teams, the communication culture, and existing work processes. Similarly, participants mentioned that teams striving to prioritize contextual care may be constrained by systemic barriers, such as fragmented information systems or financial structures that favour treatment volume over appropriateness.

These interconnected influences highlight the importance of a multilevel approach to implementation, in which supportive conditions at one level can reinforce change at another. We found that the factors followed a logical order in how they influenced behaviour:

1. *Is contextualizing care perceived as important?* (Motivation)
2. *Do HCPs consider themselves capable of contextualizing care?* (Capability)
3. *Is contextual care supported within their clinical environment?* (Opportunity)

Although these elements are not strictly linear, this progression reflects the dominant dynamic observed in our data. These findings suggest that even when professionals recognize the value of contextualized care, systemic and environmental conditions often determine whether that care is actually delivered in practice.

### Possible intervention strategies to improve contextualizing care

To support the implementation of contextualized care, we linked the nine identified factors to the intervention functions from the BCW (see Table 3). Because these factors were already categorized according to the COM-B components, their connection to the corresponding BCW intervention functions followed logically. The specific intervention suggestions presented in Table 3 were developed by the research team. While they were informed by participants’ perspectives and experiences, they do not stem directly from the focus group data but rather reflect example strategies based on the researchers’ interpretation.

**Table 3:** Intervention suggestions based on the identified factors

COM-B component	No	Factor	Intervention types	Example strategies
**Capability** (psychological and physical)	1	Skills and knowledge to engage with relevant patient context	Education Enablement Training	Provide training on contextual communication and cultural sensitivity Integrate contextual factors into clinical guidelines or protocols Use coaching on the job for workplace learning on contextual care
**Opportunity** (physical)	2	Supportive environment for contextual conversations	Enablement Restriction Environmental restructuring	Integrate contextual factors into history taking forms Introduce longer consultation slots for complex patients Promote low-distraction settings (e.g. phone-free consultations, preparatory review of records, private rooms)
**Opportunity** (physical)	3	Fragmented information systems hinder contextual care	Enablement Restriction Environmental restructuring	Standardize contextual fields in electronic health records (EHRs) Improve system interoperability across disciplines Use prompts or flags to update contextual data
**Opportunity** (physical)	4	Systemic drivers that misalign with contextual care	Enablement Restriction Environmental restructuring	Adjust reimbursement to require contextual assessment for intensive treatments Include contextual criteria in care quality frameworks and audits
**Opportunity** (social)	5	Lack of shared team norms around contextual care	Enablement Restriction Environmental restructuring	Facilitate team reflection sessions on contextual care Appoint contextual care ambassadors Embed shared norms in team onboarding or protocols
**Opportunity** (social)	6	Collaboration and communication across healthcare professionals	Enablement Restriction Environmental restructuring	Develop structured handover tools including relevant patient context Establish protocols for transmural collaboration (e.g. with GPs) Create space for interprofessional dialogue
**Opportunity** (social)	7	Trust and emotional complexity in patient interactions	Enablement Restriction Environmental restructuring	Provide peer support, debriefings, or moral case deliberation Offer supervision on navigating emotionally complex care situations
**Motivation** (automatic)	8	Intrinsic motivation to provide person-centred care	Enablement Environmental restructuring Modeling Persuasion Coercion	Highlight positive examples in team meetings Reinforce contextual care in team vision, evaluation, and feedback loops
**Motivation** (reflective)	9	Consequences of overlooking or integrating relevant patient context	Education Persuasion Coercion Incentivization	Use real-life patient stories to show the impact of missed context Collect evidence on improved outcomes from contextualized care

Table 3 shows that the nine identified factors span all COM-B components. This implies that a broad multifaceted implementation approach is needed. However, most barriers were concentrated in the Opportunity component, particularly those related to environmental conditions such as fragmented information systems and social aspects such as lack of shared team norms and interprofessional collaboration. Therefore, the biggest accelerator for increased uptake of the desired behaviour (“signaling, exploring, and integrating the life context of patients with a serious illness into care provision and planning by HCPs”) might be addressing these environmental conditions [31]. A key challenge is that these changes should be facilitated by policy-level decisions and system-level changes beyond the direct control of individual HCPs. Therefore, sustainable improvement in contextual care should depend not only on individual capability and motivation but also on broader team and system support.

## Discussion and conclusion

### Discussion

This study identified key factors influencing the delivery of contextualized care for patients with serious illness. While participants were motivated and generally confident in their communication skills and ability to understand patient context, they encountered substantial individual, team, and system barriers. These included training gaps—especially regarding sociocultural competence and practical application—alongside environmental constraints such as time pressure, fragmented information systems, and lack of shared norms. Such barriers often outweighed motivation and capabilities, particularly in complex or acute settings. Systemic drivers, including financial incentives and societal expectations, further reinforced a biomedical focus. The challenge is therefore less about willingness than feasibility, underscoring the need for multilevel strategies that address organizational and systemic barriers while supporting individual capability.

### Understanding relevant context: from concept to practice

Although participants valued the importance of patient context, not all distinguished between general context and *relevant* context—i.e. factors that directly influence the appropriateness or feasibility of care. This conceptual gap can lead to care that is misaligned with patients’ real-life circumstances and may reduce adherence [3, 5, 20, 42]. Many participants associated “context” primarily with sociocultural aspects, potentially overlooking practical or existential factors such as living arrangements, caregiving responsibilities, or personal values, which may be more decisive in serious illness [43, 44]. Existing communication models, such as Calgary-Cambridge, promote biopsychosocial awareness [45–47] yet often emphasize the process of communication over its substantive content. This focus may foster a false sense of competence, while the skill to identify and integrate relevant contextual information into care planning remains underdeveloped [5]. Furthermore, participants described a disconnect between what is taught in formal training and what is reinforced in daily practice [48]. Contextualizing care is often learned through experience rather than instruction [5, 29]. Workplace-based learning, team reflection, and interprofessional coaching could be promising strategies to bridge this gap [49, 50].

### Environmental and team-level barriers outweigh the individual motivation

Although professionals were motivated, they frequently described contextual care as difficult to deliver in high-pressure environments. Time constraints, limited privacy, and a lack of supportive infrastructure were seen as major barriers, especially in acute settings. Similar multilevel barriers have been observed internationally, all of which can result in non-beneficial treatment decisions, especially in the palliative phase [51–54].

In this regard, electronic health records were a major source of frustration. Participants described fragmented documentation, lack of interoperability, and inconsistent use of language. This hampered continuity and increased cognitive load. Technological solutions, such as artificial Intelligence (AI) to filter relevant context from records, may help, but relational factors are equally critical [55, 56]. Accordingly, interprofessional collaboration emerged as a key enabler and a frequent barrier. A lack of shared norms, unclear responsibilities, and hierarchical dynamics often led to fragmented care. Nurses in particular noted that their contextual observations were not always acknowledged or acted upon by physicians. Similar concerns have been reported before in interprofessional communication [57].

### From individual responsibility to shared systemic support

The findings underscore that contextualized care cannot rely on individual skills or motivation alone. Most barriers identified in this study are located in the opportunity component of the COM-B model, indicating that environmental and structural changes are essential. This includes creating time and space for contextual conversations, improving information systems, and cultivating a team culture that values contextual input in the care of patients with serious illness. Team-based reflection and routine collaboration could be particularly effective in enhancing contextual awareness and strengthening relational trust. Studies show that working in supportive, well-functioning teams not only improves care quality but also reduces burnout and enhances job satisfaction, especially among nurses [58, 59]. This study also points to a gap in existing contextual care research. While Weiner’s model identifies cognitive steps for contextualization [5], less is known about how interprofessional collaboration, communication, and documentation practices shape the ability to apply these steps in complex real-world settings. Further research should explore how teams can collectively support contextual decision-making—especially in high-turnover, acute, or fragmented care environments.

### Strengths and limitations

A key strength of this study is the use of both the TDF and COM-B models, which provide a structured foundation for the interview process and allow for in-depth analysis of the findings. In addition, applying the BCW allows us to translate these insights into focused, theory-informed recommendations, bringing the analysis full circle from factors to actionable intervention strategies. Additionally, the diversity in participants' disciplines, specialisms, hospital type, age, and work experience contributed to the richness of the data. The sample was predominantly female (16 of 20), which may have influenced the perspectives shared.

Several limitations should also be acknowledged. First, the study was conducted in two Dutch hospitals, which may limit the transferability of the findings to other healthcare systems or cultural settings. Second, although focus groups encouraged interaction among participants, some individuals may have felt less comfortable speaking freely, especially in mixed-discipline groups. Returning transcripts for member checking may have mitigated this to some extent. Psychosocial and spiritual care professionals were not included, despite their important role in eliciting the relevant patient context [60].

Finally, as participants generally reported a high affinity for contextualizing care, selection bias or social desirability bias cannot be excluded. Future research could address these concerns by using anonymous one-to-one interviews, which may elicit more nuanced or divergent perspectives, and by conducting anonymous survey studies to further substantiate and complement the factors identified in this study.

## Conclusions

HCPs are motivated and possess foundational communication skills to provide contextualized care for patients with serious illness, but environmental, team, and systemic barriers—especially those within the *Opportunity* component—limit their ability to do so. Targeted training in identifying and applying relevant patient context is essential groundwork. However, cultural change and systemic support are critical to ensure these skills can be consistently applied in practice and to align care with patients’ lived realities.

### Practice implications

Altogether, these insights underscore the urgency of shifting from individual intent to collective norms in order to better serve patients and optimize resource use. Practical steps include the following:

- Improving contextual awareness and evidence-based practice by investing in continuous education
- Embedding contextual care into workplace learning and reflective practice
- Clarifying norms around contextual care within the team
- Adapting workflows and documentation to include relevant context for patients with serious illness
- Emphasizing contextualization in quality metrics and reimbursement models

## Supplementary Information

Supplementary Material 1: Supplement 1. The interview guide for the focus groups.

Supplementary Material 2: Supplement 2. COM-B/TDF definitions for the target behaviour.

Supplementary Material 3: Supplement 3. COREQ checklist.

## Data Availability

Metadata from this study will be shared on the Radboud Data Repository (RDR) under [Collections - Published] (https:/data.ru.nl/collections/published). We are currently applying for a collection to host the metadata and will notify BMC Medicine once it becomes publicly available. The raw qualitative focus group data contain sensitive, identifiable information and cannot be shared publicly due to confidentiality agreements approved by the ethics board. To ensure transparency while protecting privacy, de-identified excerpts may be made available upon reasonable request to qualified researchers via (mailto:onderzoek.anes@radboudumc.nl), subject to confidentiality agreements.

## Abbreviations

ACP: Advance care planning
AI: Artificial intelligence
BCW: Behaviour Change Wheel
COM-B model: Capability, Opportunity, Motivation–Behaviour model
COREQ: Consolidated criteria for reporting qualitative research
HCP: Healthcare professional
MCC: Multiple chronic conditions
SDM: Shared decision-making
TDF: Theoretical Domains Framework
