# Supplementary material for: Factors impacting the delivery of contextualized care in serious illness: a focus group study with healthcare professionals
Source: BMC Med. 2026 Jan 29;24:117. doi: 10.1186/s12916-026-04662-w (PMC12924310; doi:10.1186/s12916-026-04662-w)
Supplement: Supplementary file 1 — Supplementary Material 1: Supplement 1. The interview guide for the focus groups. [file 12916_2026_4662_MOESM1_ESM.pdf]

Supplement 1: The interview guide for the focus groups

1. What skills or knowledge do you need to identify, explore, and integrate relevant patient context into care planning?
    a. How do you recognize relevant context in patient care?
    b. How do you address relevant patient context in patient care?
    c. What do you need to (better) integrate attention to the patient's context into your work practice?

2. How does your environment influence your attention to relevant patient context? In what way?
    a. Which resources (or lack thereof) influence your decision to pay attention to a patient's context? In what way?
    b. Which people in your work environment influence your decision to pay attention to a patient's context? In what way?

3. How motivated are you to pay attention to relevant patient context? Why is that?
    a. What is your expectation or experience of what happens when you pay attention to the patient's context?
