# Supplementary material for: Factors impacting the delivery of contextualized care in serious illness: a focus group study with healthcare professionals
Source: BMC Med. 2026 Jan 29;24:117. doi: 10.1186/s12916-026-04662-w (PMC12924310; doi:10.1186/s12916-026-04662-w)
Supplement: Supplementary file 2 — Supplementary Material 2: Supplement 2. COM-B/TDF definitions for the target behaviour. [file 12916_2026_4662_MOESM2_ESM.pdf]

Supplement 2: COM-B/TDF definitions for the target behaviour

COM-B components	TDF domain	Domain definition related to contextualizing care
**Capabilities**	1. Knowledge	Knowing the relevance of the context of a patient with serious illness for their care planning, the communicative process to explore it and specific domains to look for.
	2. Skills	Having the communicative and interpersonal competences to recognize contextual red flags, probe for relevant contextual domains and integrate necessary patient context in care planning.
	3. Social/professional role and identity (Self-standards)	Deciding on the basis of implicit or explicit beliefs that attention to a patient's life context is part of their professional behavior.
	4. Beliefs about capabilities (Self-efficacy)	Being confident about their abilities to perform the necessary communicative steps to contextualize a seriously ill patient's care.
	5. Behavioral regulation	The personal and organizational change process necessary to contextualize care.
	6. Memory, attention and decision processes	The ability to meet the cognitive demands necessary to contextualize care.
**Opportunities**	7. Environmental context and resources (Environmental constraints)	Material or immaterial assets impacting the ability to contextualize care.
	8. Social influences (Norms)	People who influence a health care provider's decision to contextualize care.
**Motivation**	9. Beliefs about consequences (Anticipated outcomes/attitude)	Assessing the (expected) results of contextualizing care.
	10. Emotion (Emotion)	Personal feelings influencing contextualizing care.
	11. Reinforcement	Indicating expected consequences that impact their tendency to contextualize care.
	12. Intentions	Level of commitment to contextualizing care.
	13. Goals	Imagined outcomes that a health care provider wants to achieve by contextualizing care.

	14. Optimism	Feeling confident that contextualizing care will yield positive results.
